# Risk Factors for Hepatotoxicity Due to Paracetamol Overdose in Adults

**DOI:** 10.3390/medicina57080752

**Published:** 2021-07-25

**Authors:** Iwona Popiolek, Piotr Hydzik, Pawel Jagielski, Monika Zrodlowska, Karol Mystek, Grzegorz Porebski

**Affiliations:** 1 Toxicology Clinical Department, University Hospital in Krakow, Jakubowskiego 2, 30-688 Krakow, Poland; iwona.popiolek@uj.edu.pl (I.P.); piotr.hydzik@uj.edu.pl (P.H.); 2 Department of Toxicology, Jagiellonian University Medical College, Jakubowskiego 2, 30-688 Krakow, Poland; 3 Department of Nutrition and Drug Research, Faculty of Health Science, Jagiellonian University Medical College, Skawińska 8, 31-066 Krakow, Poland; paweljan.jagielski@uj.edu.pl; 4 Faculty of Medicine, Jagiellonian University Medical College, sw. Anny 12, 31-008 Krakow, Poland; monika.orlowska33@gmail.com (M.Z.); karolmystek@gmail.com (K.M.); 5 Department of Clinical and Environmental Allergology, Jagiellonian University Medical College, Botaniczna 3, 31-503 Krakow, Poland

**Keywords:** paracetamol, acetaminophen, drug overdose, hepatotoxicity, liver injury, risk factor

## Abstract

*Background and Objectives*: Over-the-counter availability and a good safety profile make paracetamol one of the most common analgesics in developed countries but also the leading cause of liver failure due to overdose. The objectives of the study were to identify modifiable risk factors for severe hepatotoxicity following paracetamol overdose in adults. *Materials and Methods*: A retrospective cohort study involved the consecutive adult patients hospitalized in a toxicological center over a period of seven years due to paracetamol overdose. Complete medical datasets of laboratory and anamnestic variables were analyzed and validated by means of logistic regression model. *Results*: A total of 185 patients entered the study, including 25 individuals who developed severe hepatotoxicity (plasma aminotransferases levels above 1000 UI/L) and 31 individuals with mild to moderate liver injury (plasma aminotransferases levels above upper normal range, but below 1000 UI/L). In the univariable analysis, significant hepatotoxicity risk factors were male gender, alcohol abuse, an ingested paracetamol dose, and a timespan from ingestion to hospital admission. The later one was the only significant risk factor in the multivariable model (adjusted odds ratio 1.08; 95% CI: 1.03–1.12). *Conclusions*: A delay in hospital admission, resulting in a delayed administration of disease-specific treatment outweighs any other known risk factors of paracetamol-induced hepatotoxicity.

## 1. Introduction

Due to wide availability, paracetamol (acetaminophen) is frequently overdosed and used for suicide attempts. Thus, poisoning with paracetamol is the most common cause of acute liver failure in developed countries [1,2] and one of the most investigated types of drug-induced hepatotoxicity [3,4]. In paracetamol poisoning, severe hepatotoxicity is traditionally defined as alanine aminotransferase (ALT) elevation of >1000 IU/L [1,3]. Intoxicated patients are usually transported to toxicology centers, where they receive N-acetylcysteine (NAC) to prevent liver injury. This treatment is administered based on the Rumack-Matthew nomogram [5,6], which plots plasma paracetamol levels against the time since ingestion. Despite a widely available antidote therapy (NAC) and recognition of numerous risk factors, which have been discussed in the literature [7,8,9,10], acetaminophen-induced hepatotoxicity still occurs.

In this study, we aimed to investigate which clinical and laboratory parameters available in the emergency setting are the most significant risk factors for hepatotoxicity from the perspective of daily practice. To do so, we conducted a retrospective cohort study on a representative group of patients hospitalized due to paracetamol overdose. Apart from the laboratory parameters, which are applied to define and manage this clinical condition, we comprehensively analyzed with a multivariable statistical model, a clinical dataset derived from the patients’ medical histories. That way, we attempted to identify clinical features that might serve as modifiable risk factors for development of hepatotoxicity. The study findings provide learning points for practitioners in preventing the most important cause of liver failure in developed countries.

## 2. Materials and Methods

This retrospective study included a cohort of patients hospitalized in our tertiary reference center due to paracetamol poisoning over the period of recent 7 years. The center is a part of the University Hospital and consists of a toxicological ward, a specialized laboratory operating 24 h a day, and a regional information center for toxicological emergencies. It takes care of a population of more than 4.5 min. The study involved all consecutive patients who met the following inclusion criteria: (i) males and females, 18 years of age and older; (ii) informed consent obtained from the participant; (iii) discharge from hospital with a principal diagnosis coded as T39.1 according to International Statistical Classification of Diseases and Related Health Problems (ICD-10), which is used to code paracetamol toxicity. The subjects were not considered eligible for the study if they meet exclusion criteria: (i) patient younger than 18 years; (ii) inability to provide informed consent; (iii) discharge from hospital with a principal diagnosis other than T39.1, despite an initial suspicion of paracetamol poisoning. The NAC treatment was administered intravenously immediately after admission, as follows: 150 mg/kg for 15–60 min, next 50 mg/kg for 4 h, and then 100 mg/kg for 16 h.

A clinical dataset extracted from patients’ medical records included demographic and anthropometric characteristics, the results of physical examination, a timespan from ingestion to hospital admission (determined on basis of patients’ statements and/or patients’ relatives or caregivers statements), a dose of ingested paracetamol (determined on basis of patients’ statements and/or patients’ relatives or caregivers statements), information on coingested substances and alcohol abuse (based on anamnesis and psychological/psychiatric consultation), the results of routine laboratory tests, plasma paracetamol levels, and plasma aminotransferases levels at specific time points (on admission as well as 24 and 48 h after admission). For further analysis, patients were classified based on the highest recorded aminotransferases levels into three groups: (A) no hepatotoxicity (aspartate aminotransferase (AST) and ALT within a normal range), (B) mild to moderate liver injury (AST or ALT within 41–1000 IU/L), and (C) severe hepatotoxicity (AST or ALT > 1000 IU/L). The estimated paracetamol concentration at the 4th hour after intoxication ([PRC]_4h_) was also analyzed, since, at this time point, absorption of the ingested paracetamol is considered to be completed and it is the starting point of the Rumack-Matthew nomogram. The values were calculated according to the formula [PRC]_4h_ = [PRC]_pl_/2e^−(0.693/4)t^ given by Waring et al. [11] ([PRC]_pl_, plasma paracetamol concentration at admission).

Statistical methods: The results were reported as number (percentage), mean (standard deviation), or median (interquartile range [IQR]), where applicable. Assignment to particular groups (A, B and C) was considered as an ordinal variable. Quantitative variables were compared with the Kruskal–Wallis analysis of variance, followed by post hoc tests for significant differences. Associations of categorical variables were tested with the chi-square test and univariable logistic regression. The significance level α was set at 0.05. Subsequently, selected variables were validated and entered into a multivariable logistic regression model to check their association with severe hepatotoxicity. The statistical analysis was performed using Statistica (data analysis software system), version 13, TIBCO Software Inc., 2017, Tulsa, OK, USA. The figures were prepared using OriginPro, Version 2020b, OriginLab Corporation, Northampton, MA, USA.

## 3. Results

### 3.1. Patient Characteristics

A total of 185 patients (median age, 24 [IQR, 19–36] years; 116 women [63%]) were identified as eligible for analysis following the above criteria. The most frequent symptoms on admission were nausea and/or vomiting (*n* = 123; 66%), abdominal pain (*n* = 112; 60%), and altered mental status (*n* = 37; 20%). Suicidal ideation was reported by 70 patients (41%). Alcohol abuse was noted in 40 patients (21.6%). Alcohol blood levels were determined in 102 (out of 185) patients. Twenty-one of them had detectable ethanol concentration (range: 0.03–2.98‰, mean 1.39‰). Severe hepatotoxicity was reported in 25 patients (group C), and mild to moderate liver injury in 31 patients (group B). Quantitative clinical and laboratory variables characterizing groups A, B, and C are presented in Table 1. The treatment with NAC was administered in 181 individuals. In the remaining four cases, NAC was not given because of the very low plasma acetaminophen level measured early after the ingestion. No deaths were reported during hospitalization in the studied group. No severe symptoms of encephalopathy, corresponding to grade III or grade IV according to West-Haven Criteria [12], were observed in the study group. Altered mental status was observed at admission in 26 patients. In all cases, it was related to ethanol or coingestion of other drugs (Supplementary Table S1) and had improved when substances eliminated. Taking into account criteria involving coagulation disturbances and jaundice [13], two patients were diagnosed with acute liver failure. Both did not meet Kings College Criteria for liver transplantation [14] and had good clinical outcomes. One patient from group B had chronic B hepatitis, and one from group C had chronic C hepatitis. No other data on chronic liver failure have been reported in the study group. During paracetamol poisoning, 43% of patients also took other drugs (48% in the group with hepatotoxicity and 42% in the rest of the patients, the difference was not statistically significant). 52.4% of these were over-the-counter medications. Analgesics were the most common group (no significant statistical difference between patients with and without hepatotoxicity: 16% vs. 35%, respectively). The detailed list of reported drugs is provided in Supplementary Table S1.

### 3.2. Comparison between the Groups

Although the majority of the entire study group were women, men predominated in group C (severe hepatotoxicity). Per definition, aminotransferases plasma levels were significantly higher in groups with liver injury and hepatotoxicity (groups B and C). As shown in Table 1, the values of variables: “Paracetamol dose” and “Time from ingestion to hospital admission”, but not “Age” or “Body mass index” were on a significant increase in groups B and C. Plasma paracetamol concentration at admission did not differ between groups, but the estimated paracetamol concentration at the 4th hour after intoxication was significantly higher in group C in comparison to group A (Table 1). As expected, routine laboratory parameters reflecting liver injury (plasma bilirubin level, international normalized ratio, and platelet count) demonstrated the same pattern of blood levels as aminotransferases, whereas the results of other standard laboratory blood tests (hemoglobin level, leukocyte count, creatinine concentration) did not differ significantly between groups A, B, and C (Table 1). Neither smoking status nor suicidal intention showed significant statistical differences between patients with and without hepatotoxicity (24% vs. 43%, and 44% vs. 40%, respectively). Increased ethanol blood concentrations were recorded in 17 patients in group A, 2 patients in group B, and 2 patients in group C (mean values 1.4‰, 1.08‰, 1.5‰ respectively). The differences were not statistically significant.

**Figure 1:**
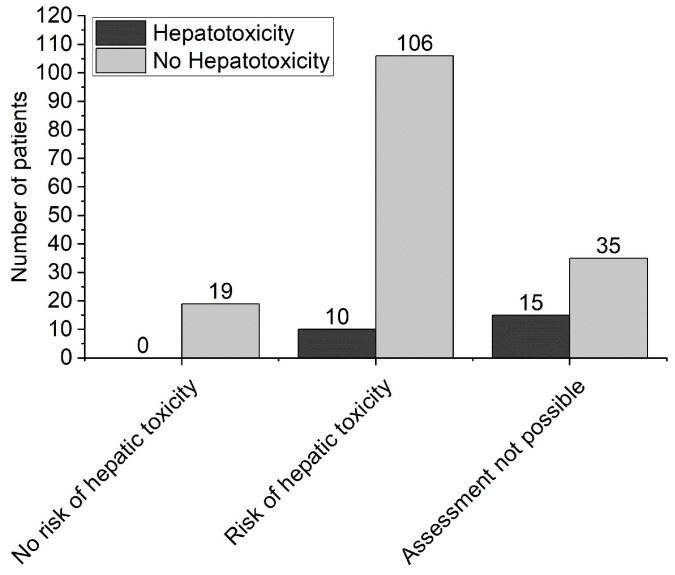
The relationship between assignment to hepatotoxicity risk assessed by means of the Rumack-Matthew nomogram (“risk”, “no risk” and “assessment not possible” on the horizontal axis) and hepatotoxicity (group C), determined on the basis of plasma aminotransferases measurement.

The relation between assignment to hepatotoxicity risk (assessed by means of the Rumack-Matthew nomogram) and hepatotoxicity (group C), determined on the basis of plasma aminotransferases measurement, is presented in Figure 1. No patients classified as “no risk” using the nomogram developed hepatotoxicity. The other 10 patients classified as being at risk of hepatotoxicity with the nomogram showed aminotransferase activity over 1000 IU/L, despite specific treatment. Finally, of the 50 patients in whom the risk could not be established with the nomogram, 15 (30%) developed hepatotoxicity.

**Figure 2:**
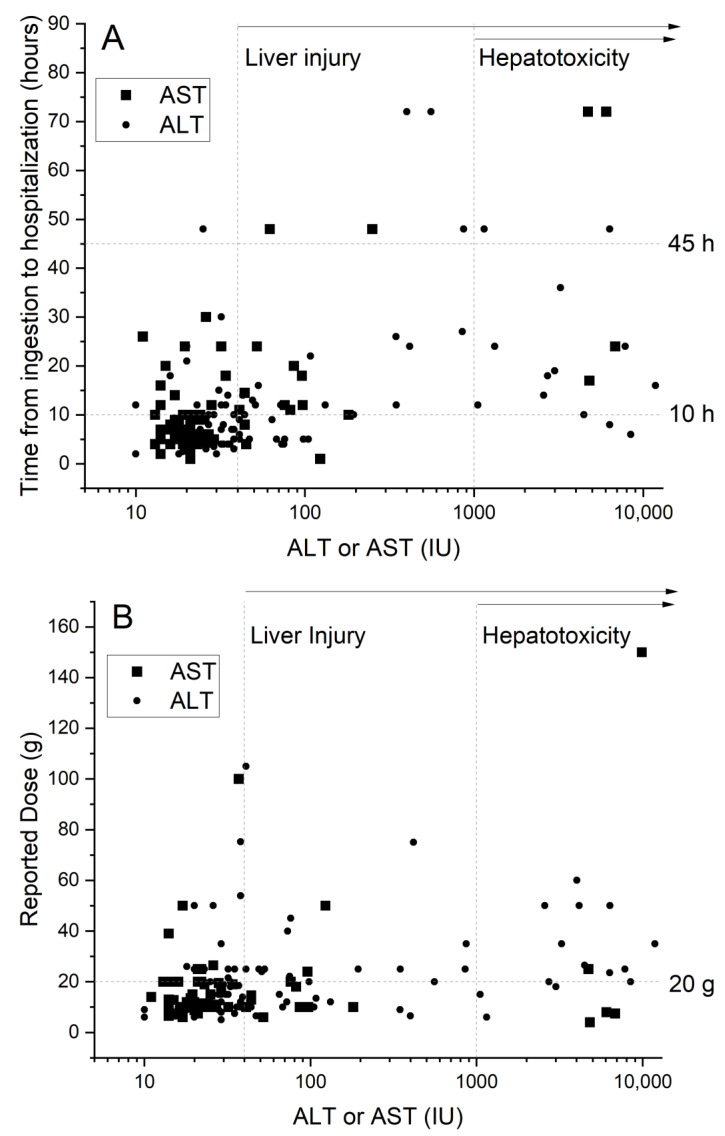
The relationship between the asparagine aminotransferase (AST) or/the alanine aminotransferase (ALT) levels and time from paracetamol ingestion to hospitalization (**A**). The relationship between AST/ALT and the reported paracetamol dose (**B**). Squares and circles represent AST and ALT values, respectively. The higher value among ALT and AST is displayed. The values on X axes are presented on a log 10 scale.

The relationship between AST or ALT levels, and time from paracetamol ingestion to hospitalization is shown in Figure 2A. Similarly, the relationship between AST or ALT levels, and the reported paracetamol dose is presented in Figure 2B. These plots can help estimate the cutoffs that are significant for increased risk of liver injury (group B) or severe hepatotoxicity (group C). Therefore, for instance, all but three severe hepatotoxic patients were admitted 10 h or more after taking paracetamol, while of the remainder of the study group, most were admitted at a shorter time. A similar interpretation can be used for the paracetamol dose (Figure 2B), although the proposed cut-off of 20 g has a lower discriminant value between patients with and without severe hepatotoxicity.

### 3.3. Logistic Regression Analysis

Uni- and multivariable logistic regression models were applied to determine the variables associated with hepatotoxicity after paracetamol overdose. For this purpose, dichotomous outcomes had to be applied. In this analysis, they were: (i) patients who developed severe hepatotoxicity (group C), and (ii) the remaining patients (groups A and B). The selected quantitative variables listed in Table 1, together with categorical variables (“gender” and “alcohol abuse”), were analyzed to develop the model. The variables “ALT level” and “AST level” did not enter the model, as hepatotoxicity is defined by them. In the univariable analysis, significant qualitative risk factors were male gender and alcohol abuse, whereas significant quantitative risk factors were the ingested acetaminophen dose and a timespan from ingestion to hospital presentation (Table 2). The latter was the only significant variable in the multivariable model (the adjusted odds ratio with the confidential interval is provided in Table 2).

## 4. Discussion

Considering the main clinical features, our study group was comparable to other cohorts of patients with paracetamol intoxication. The median age of our patients (24 years) corresponded to the values reported by Chomchai et al. [15] (23 years) and Marks et al. [2] (31 years). In addition, a slight predominance of female patients was reported previously (65.8–66.6%) [11,16]. Suicidal ideation was declared by 39% of patients in a study by Hawton et al. [17], as compared with 41% in our study. The most frequent symptoms on admission, such as nausea, vomiting, and abdominal pain, were also typical for this clinical condition [5]. Alcohol abuse was noted in almost a quarter of our patients (21.6%), as compared with 33% of patients with alcohol-related diagnoses in a similar study [18]. Patients who took additional medications during paracetamol poisoning constitute a significant part of the study group (43%), but the detailed impact of concomitant medications on hepatotoxicity is difficult to assess due to the wide variety of these drugs (Supplementary Table S1).

For a more detailed assessment of the risk factors for hepatotoxicity, and in view of the fact that a considerable proportion of patients presented with a moderate increase in plasma aminotransferase levels (over the normal range but below 1000 IU/L), we divided the study population into three groups, as described above. In the available literature, there are numerous studies that have discussed new risk factors for hepatotoxicity [9,10,11,15,16,19,20,21,22,23,24], including parameters related to the patient’s medical history, acetaminophen dose, biochemical findings, or a combination thereof. Quantitative and qualitative medical variables, which might serve as such risk factors in the studied population, are presented in Table 1 and Table 2.

As expected, the paracetamol dose appeared to be a significant risk factor for hepatotoxicity, and its significance was shown to be even greater than that of a paracetamol dose per kilogram of body mass, as body mass index itself was not a risk factor (Table 1). However, utility of a paracetamol dose as a risk factor is limited by the fact that it is difficult to precisely estimate the absorbed amount of the drug. Due to that, the measurement of plasma paracetamol levels in patients may be of interest. In our study, the plasma level of paracetamol at admission did not differentiate between groups A, B, and C in regard to the hepatotoxicity risk. This may be caused by variability in the time periods between paracetamol ingestion and blood sampling for the measurements. However, the modified variable (an estimated paracetamol concentration at the 4th hour after intoxication) turned out to have only slightly better performance (Table 1). The next statistically significant risk factor was “time from ingestion to hospital admission” followed by NAC treatment. This finding is in line with the results of other authors who reported that early NAC administration is related to a low rate of hepatotoxicity in patients [25,26]. Data related to parameters derived from patient statements (especially in case of alcohol abuse) might be inaccurate, which is a limitation of the study. Surprisingly, age in our study did not turn out to be a statistically significant risk factor, although a trend towards older age was observed in groups with more severe hepatotoxicity (Table 1). This may be related to the fact that although age 40 years or above was identified as a significant independent risk factor for fulminant hepatic failure due to paracetamol poisoning in another group of patients [27], in our study group, there were few patients with such a severe clinical course.

As mentioned in the introduction, the assessment with the Rumack-Matthew nomogram determines further steps with a patient after paracetamol overdose [5,28]. However, in some clinical situations (e.g., ongoing or delayed paracetamol absorption, an unknown time period from overdose, an ingestion of paracetamol at several separate doses), utility of the nomogram is very limited or simply impossible. Indeed, 50 of our patients were initially classified as “unknown risk” with the nomogram and 30% of them subsequently developed hepatotoxicity (Figure 1), which prompts the search for independent risk factors of hepatotoxicity. This was just the goal of the final part of the analysis by means of logistic regression.

The univariable analysis showed that apart from the above discussed variables (a paracetamol dose and timespan from ingestion to hospitalization), two others, namely male gender and alcohol abuse, were significant risk factors for hepatotoxicity. A possible explanation is that men were expected to be late in seeking medical attention. This can also apply to alcohol abusers. In addition, they may demonstrate a deterioration in liver function with glutathione depletion because of underlying chronic medical conditions [29]. Thus, both of the above variables might be affected by a delay in hospital admission, which was identified as the most significant risk factor of paracetamol-induced hepatotoxicity, overweighing the effect of all other variables included in the final analysis by means of the multivariable model.

## 5. Conclusions

To conclude, this comprehensive analysis in a large group of adults, supported by a multifactorial statistical model, showed that delay in hospital admission, resulting in delayed NAC administration, is associated with the highest risk of paracetamol-induced hepatotoxicity. This main conclusion should not obscure the fact that individual risk factors that are difficult to capture in the overall analysis of the study results may still play a significant role in particular patients. For instance, during intoxication, a significant number of patients took other drugs in addition to paracetamol, but due to their wide variety in individual cases, reliable assessment of their effects by means of systematic statistical analysis is difficult. The novelty of our study is that it provides strong evidence that the importance of this risk factor outweighs any other known risk factors. This finding has direct implications for daily clinical practice, as it shows that hospital admission and administration of specific treatments should be prioritized over other procedures.

## Figures and Tables

**Table 1:** Quantitative clinical and laboratory variables characterizing groups A, B, and C.

	Group A (129 Patients, 92/37 F/M)	Group B (31 Patients, 15/16 F/M)	Group C (25 Patients, 9/16 F/M)	*p*
Laboratory parameters				
ALT, IU/L	19 (10)	137 (231)	2464 (2657) *	<0.001
AST, IU/L	20 (8)	83 (99)	3669 (5675) *	<0.001
[PRC]_pl,_ mg/L	101.1 (105.2)	76.3 (78.5)	102 (104.3)	0.57
[PRC]_4h,_ mg/L	169 (153)	239 (322)	373 (249) *	0.11
Bilirubin, µmol/L	13 (10)	16 (10)	67 (117) *	<0.001
INR	1.15 (0.15)	1.14 (0.14)	1.79 (0.77)	<0.001
Platelet count, 103/mL	240 (60)	250 (62)	181 (67) *	<0.001
Hemoglobin, g/dL	13.1 (1.7)	13.3 (1.9)	13.2 (2.2)	0.70
Leukocyte count, 103/µL	8 (3.2)	9.1 (3.5)	8 (3.2)	0.39
Creatinine, µmol/L	64 (19)	67 (16)	117 (168)	0.16
**Clinical features**				
Paracetamol dose, grams	17.00 (15.58)	23.12 (15.44) *	31.50 (31.45) *	<0.001
Time from ingestion to hospital admission, hours	8 (7)	20 (19) *	30 (22) *	<0.001
Age, years	28 (15)	30 (14)	32 (15)	0.40
BMI, kg/m^2^	23.4 (4.5)	25.6 (5.8)	25 (5)	0.15

*p* Values were derived from Kruskal-Wallis ANOVA test. Data are presented as mean (standard deviation). * *p*-values < 0.05 in post hoc tests for ANOVA for comparison between group A and group B or C. Abbreviations: ALT, alanine aminotransferase; AST, aspartate aminotransferase; BMI, body mass index; F/M, female/male; [PRC]_pl_, plasma paracetamol concentration at admission; [PRC]_4h_, estimated paracetamol concentration at 4th hour after intoxication; INR, international normalized ratio.

**Table 2:** Univariable and multivariable analysis of risk factors for hepatotoxicity during hospitalization after paracetamol overdose.

Variables	Univariable Analysis		Multivariable Analysis	
	OR	95% CI	Adjusted OR	95% CI
Male gender	2.94	1.24–6.99	1.85	0.35–9.88
Alcohol abuse	3.55	1.46–8.61	3.59	0.69–18.66
Age, years	1.01	0.99–1.04	0.99	0.94–1.05
BMI, kg/m^2^	1.05	0.96–1.15	1.04	0.88–1.23
Paracetamol dose, grams	1.03	1.01–1.05	1.02	0.98–1.07
Time from ingestion to hospital presentation, hours	1.06	1.03–1.09	1.08	1.03–1.12

Statistically significant results are underlined. Abbreviations: BMI, body mass index; CI, confidential interval; OR, odds ratio.

## Data Availability

Data supporting reported results can be found in the Jagiellonian University Repository at https://ruj.uj.edu.pl/xmlui/ (accessed on 24 July 2021).

## Supplementary Materials

The following are available online at https://www.mdpi.com/article/10.3390/medicina57080752/s1, Table S1: Other drugs coingested during paracetamol poisoning.
