# Supplementary material for: Risk Factors for Hepatotoxicity Due to Paracetamol Overdose in Adults
Source: Medicina (Kaunas). 2021 Jul 25;57(8):752. doi: 10.3390/medicina57080752 (PMC8402128; doi:10.3390/medicina57080752)
Supplement: Supplementary file 1 [file medicina-57-00752-s001.zip › medicina-1273273-supplementary.pdf]

Supplementary Table S1. Other drugs coingested during paracetamol poisoning.

Name of drug or drug class	Number of cases	Name of drug or drug class	Number of cases
Ibuprofen	24	Benzodiazepines and Z-drugs*	15
Aspirin	6	Tramadol	11
Other NSAIDs	8	Antihistaminics	9
Dextromethorphan	12	Antidepressants	8
Pseudoephedrine	8	Codeine	7
Herbal drugs	5	Antipsychotics	5
Caffeine	3	Antiepileptics	3
Others	19		

*Zolpidem or Zopiclone
